# Exploring Venue-Associated Risk: A Comparison of Multiple Partnerships and Syphilis Infection Among Women Working at Entertainment and Service Venues

**DOI:** 10.1007/s10461-013-0546-5

**Published:** 2013-07-13

**Authors:** Sharon S. Weir, Jing Li, Jessie K. Edwards, Anisha D. Gandhi, Huang Yingying, Chirayath M. Suchindran, Xiang-Sheng Chen

**Affiliations:** 1 Carolina Population Center, University of North Carolina at Chapel Hill, Campus Box 8120, Chapel Hill, NC 27546 USA; 2 Department of Epidemiology, Gillings School of Global Public Health, University of North Carolina at Chapel Hill, Chapel Hill, NC 27546 USA; 3 Duke Population Research Institute and Duke Global Health Institute, Duke University, Durham, NC USA; 4 Institute of Sexuality and Gender and Department of Sociology, Renmin University of China, Beijing, China; 5 Department of Biostatistics, Gillings School of Global Public Health, University of North Carolina at Chapel Hill, Chapel Hill, NC USA; 6 The National Center for STD Control and the Chinese Academy of Medical Sciences and Peking Union Medical College Institute of Dermatology, Nanjing, China

**Keywords:** Syphilis, Social venues, Venue-based prevention, China

## Abstract

The re-emerging syphilis epidemic in China is documented among sex workers, but little is known about STI risk among the broader group of women who work at entertainment and service venues, many of whom do not self-identify as sex workers. In 2009 in Liuzhou, China, community informants identified venues where people meet sexual partners. Characteristics of a stratified random sample of venues were collected during venue visits. Female staff at 42 venues were interviewed and tested for syphilis. The results showed that venue characteristics, worker behaviors, and syphilis prevalence differed by venue type. Service venue workers had more sexual partners, were more likely to report sex work, and more likely to have a positive syphilis test than entertainment venue workers (prevalence ratio: 5.4; 95 % CI 1.4–20.6). To conclude, risk of syphilis differs by venue type and is higher at service venues, even among women who do not report commercial sex.

## Introduction

After virtual elimination between 1960 and 1980, the syphilis epidemic in China has re-emerged [1]. Prevalence of infection is high among populations known to have high rates of new sexual partnerships, such as men who have sex with men [2] and sex workers [3], but there is concern that syphilis transmission is spreading to the general population [4–6]. Among first time blood donors, the prevalence of serologic markers for syphilis increased from 0.41 % to 0.57 % over the period 2008–2010 [7]. The rate of congenital syphilis increased from 0.01 cases per 100,000 live births in 1991 to 19.68 cases per 100,000 live births in 2005 [1]. If untreated, syphilis can have significant health sequelae and increase the risk for acquisition and transmission of HIV.

Contributing to the syphilis epidemic in China are changes in the rate and pattern of new sexual partnerships so extraordinary that the shift in behavior has been described as a “sexual revolution” [8]. According to national sexual behavior surveys, the percent of women who had two or more sexual partners in her lifetime more than tripled between 2000 and 2006, increasing from 8.1 to 29.6 % [9]. Underlying these changes in partnership rates are social determinants [4, 10, 11] including an increased acceptability of pre- and extramarital sex, a booming cash economy, urbanization, a growing male-to-female ratio arising from the one-child policy [12], and an increasingly mobile labor force. The demand for sex work, which, although covertly practiced due to stigma and periodic government crackdowns, has also increased [4, 10].

As discretionary income becomes more available, public venues have gained importance as risk environments [5]. A household survey in Liuzhou, a city in southwest China, found that approximately 20 percent of sexually active adults reported meeting a current sexual partner at an entertainment venue [13]. Women in Liuzhou who had danced in an entertainment venue in the past year or had had a full body massage were more likely to have had multiple sexual partners [13]. A survey of market vendors in an eastern city in China found that visiting karaoke bars, discos or massage parlors in the past 30 days was associated with unprotected sex and sexually transmitted disease [14]. Among sex workers, the prevalence of syphilis varies by type of venue, with the highest prevalence among those who recruit outdoors (such as on the street), lower among those who work in service venues (such as massage parlors) and lowest among those who work in entertainment venues (such as karaoke bars) [15–19]. Less is known, however, about whether the prevalence of syphilis and sexual risk-taking behaviors vary by type of venue among other female workers at these venues.

The objective of this study is to describe entertainment and service venues where people meet new sexual partners in Liuzhou, China; describe the characteristics of all female workers in those venues (regardless of whether they engage in sex work); and assess whether risk behavior and syphilis infection among female workers vary by type of venue. Syphilis risk among sex workers is well-documented; information about sexual risk among the entire group of women working at entertainment and service venues is limited.

## Methods

### Identification and Characterization of Venues

We used the Priorities for Local AIDS Control Efforts (PLACE) method [20] in four urban districts and six rural counties of Liuzhou, China, to identify and characterize venues where people meet new sexual partners, and to describe people who work at those venues [21]. First, interviewers asked 402 community informants aged 18 and older to name venues where people go to meet new sexual partners, including but not limited to commercial sex partners. The 971 named venues were sorted by geographic area, type, and number of informants reporting it. Next, we selected a stratified random sample of 385 venues to visit, including one-third of urban venues named by a single informant; two-thirds of urban venues named at least twice; and one-fifth of all venues identified in the six counties surrounding urban Liuzhou regardless of the number of nominations. During a visit to 334 venues (the other 51 were closed or the manager refused entrance), interviews were conducted with an informant at each venue—usually a manager—to characterize the personnel, patrons, and activities taking place at the site.

Next we divided the 334 venues into three groups using the categories recently recommended by researchers (including co-author Chen) at the National STD Control Center [22]:

1. “Service Venues” are massage parlors, hair salons, saunas, hotels, and guesthouses. Patrons are usually male, often come alone, and have limited social interaction with other customers. Employees, who are primarily or entirely female, may welcome and socialize with patrons, provide massages, or serve drinks and snacks—this contact allows employees to negotiate sexual services with clients.
2. “Entertainment Venues” are night clubs, karaoke bars, restaurants, discos and other places where people socialize. Men and/or women often come in groups, consume alcohol, and sing or dance, sometimes accompanied by female employees.
3. “Outdoor Venues” are venues located outdoors such as street corners and parks.

As there were too few outdoor venues to analyze as a distinct category, the remainder of the analysis is restricted to the 200 service venues and 115 entertainment venues in the sample.

### Surveys of Female Workers at Entertainment and Service Venues

We selected a stratified random sample of 28 service venues and 14 entertainment venues, with oversampling of venues where sex work was reported and oversampling of venues from the four urban districts of Liuzhou. All female workers aged 15 and older at selected venues were eligible to be interviewed. Those providing verbal informed consent completed a face-to-face survey describing their socio-demographic status, work history, sexual partnerships, substance use, and HIV knowledge/testing history. The interviews were administered in Mandarin Chinese or Zhuang, the language spoken by the largest ethnic minority group in Liuzhou. Participants were also tested for evidence of syphilis using a rapid syphilis test (Wantai-anti-TP Antibody Rapid Test). A positive test reflects evidence of an antibody to syphilis and is interpreted as a lifetime marker of ever having been infected. Those with a positive test were encouraged to obtain confirmatory testing to diagnose active syphilis infection, but information on whether participants sought such testing or received a positive confirmatory test result was not available. Interviews and rapid tests were conducted within a private setting at each venue, and during off-peak hours arranged with the site manager as necessary.

No unique identifiers were obtained. A payment of 100 yuan (ca. US$ 14) was given to all who were eligible and agreed to participate in the survey and be tested. There were no direct refusals for either the survey or the syphilis test among those asked to participate; however, 58 female workers at one large urban venue left before an interviewer requested informed consent. Study staff reported that there were too few interviewers at the site, causing wait times to be much longer than usual. Study protocols were approved by the Research Ethics Committee of the National Center for STD Control, China and the Institutional Review Board at the University of North Carolina. Data were entered in Liuzhou, China and analyzed at the University of North Carolina in collaboration with the National Center for STD Control in China.

### Statistical Analysis

**Fig. 1:**
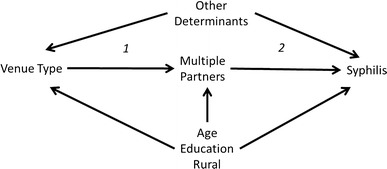
Directed acyclic graph illustrating the proposed causal pathway between type of venue and syphilis

Venue and individual-level data were weighted based on the multi-stage sampling design and probability of selection. We compared worker characteristics and the prevalence of syphilis infection at service and entertainment venues. Hypothesized causal pathways between type of venue, multiple sexual partnerships and evidence of syphilis infection, were conceptualized using directed acyclic graphs [23] (Fig. 1) and log-binomial regression was used to estimate prevalence ratios for the relationships between variables on this proposed causal pathway. These included (1) effect of the venue type on the probability of having multiple (two or more) sexual partners in the past year; (2) the effect of having multiple partners on evidence of syphilis infection; and (3) the total effect of venue type on the prevalence of a positive rapid syphilis test. We used generalized estimating equations to account for correlated outcomes among workers at the same venue. We repeated this process using linear regression to calculate prevalence differences for each contrast of interest.

## Results

### Comparison of Entertainment and Service Venue Environments

The 115 entertainment venues differed from the 200 service venues in physical characteristics, onsite activities and patronage (Table 1). Service venues were smaller than entertainment venues, employed fewer workers, had fewer patrons, and were more often located within a geographic cluster of similar venues. Almost all of the service venues reported having rooms with a bed. Activities enjoyed by groups of friends and peers, such as socializing for an hour or more, alcohol consumption, and singing karaoke were more likely to occur at entertainment venues. Entertainment venues were also more likely to be patronized by both men and women. In contrast, many service venues (34.3 %) reported having no female patrons even during the venue’s busiest time. Activities such as massage that were provided by venue staff were more likely in service venues. Approximately half of venue informants at both entertainment and service venues confirmed that people meet new sexual partners at the venue, and approximately one-fourth reported that there was someone onsite that helped potential partners meet. More informants at service venues (35.7 %) than at entertainment venues (6.6 %) reported that some female workers provided sex for money. HIV prevention activities, including HIV education talks, condom distribution, posters, and health worker outreach, were more common at service venues (69.5 vs. 42.2 %), and managers at service venues were more willing to sell condoms onsite.

**Table 1:** Characteristics of venues where people go to meet new sexual partners in Liuzhou, China

	Entertainment venues (*n* = 115) %^**a**^ (95 % CI)	Service venues (*n* = 200) %^**a**^ (95 % CI)
Total	39.1 (32.9, 45.3)	60.9 (54.7, 67.1)
Venue type, location, and years of operation		
Social venues		
KTV	29.6 (20.2, 39.0)	
Bar	22.6 (14.3, 30.9)	
Nightclub/disco	9.5 (4.0, 14.9)	
Restaurant	6.3 (0.8, 11.9)	
Internet café/bar	5.3 (0.1, 10.5)	
Karaoke	3.0 (0.2, 5.7)	
Other	9.1 (3.5, 14.8)	
*Total*	100.0	
Service venues		
Massage		46.7 (38.7, 54.6)
Hair salon		24.9 (17.9, 32.0)
Sauna		9.5 (5.0, 14.0)
Hotel		^c^
Guesthouse		18.9 (12.0, 25.7)
*Total*		100.0
Located in urban Liuzhou	66.8 (56.1, 77.4)	62.7 (55.4, 70.0)
Site is in a cluster	4.7 (1.7, 7.6)	55.0 (47.0, 63.1)
Years in operation < 1 year	13.8 (6.8, 20.8)	27.0 (19.7, 34.2)
Characteristics of staff and patrons		
Ratio of female staff to male staff > 1	31.2 (21.4, 41.1)	89.1 (83.7, 94.5)
10 or fewer patrons present during busiest time	1.7 (0.0, 4.9)	54.6 (46.5, 62.7)
More than 25 patrons present during busiest time	94.4 (89.4, 99.3)	19.5 (13.1, 25.9)
No female patrons present during busiest time	4.3 (0.0, 9.1)	34.3 (26.7, 41.9)
Ratio of male to female patrons > 1	81.1 (73.2, 88.9)	90.7 (86.0, 95.5)
Average number of female workers in past week	14.2 (11.0, 17.4)	6.0 (5.1, 6.9)
Activities occurring at venue		
People socialize for an hour or more	95.2 (90.2, 100.0)	68.1 (60.6, 75.6)
Alcohol consumption	68.4 (58.7, 78.2)	5.0 (1.4, 8.6)
People come for dancing	12.3 (6.5, 18.1)	^c^
People come for singing karaoke	50.2 (39.9, 60.4)	2.1 (0.0, 5.0)
People come for foot/body massage	5.1 (0.4, 9.9)	68.0 (60.1, 76.0)
People meet new sexual partners^**b**^	45.7 (35.5, 55.8)	54.2 (46.2, 62.1)
Someone onsite helps potential partners meet	20.1 (12.1, 28.1)	28.9 (21.8, 36.0)
Some female workers provide sex for money	6.6 (2.3, 11.0)	35.7 (28.3, 43.1)
People have sex onsite	11.8 (4.9, 18.7)	47.0 (39.1, 54.9)
Physical characteristics observed at venue		
Rooms onsite with bed	18.4 (9.7, 27.1)	90.8 (85.6, 96.0)
Poster with sexy lady promoting pleasure	4.3 (1.2, 7.5)	22.0 (15.6, 28.3)
Posters with HIV-related messages	4.7 (0.3, 9.0)	6.3 (2.6, 10.0)
HIV prevention at site		
Condom onsite and shown to interviewers	11.8 (4.4, 19.2)	36.7 (28.7, 44.6)
Ever had any HIV prevention activities on site	42.2 (32.0, 52.4)	69.5 (62.1, 76.9)
Any HIV educational talk at site	19.8 (11.3, 28.2)	38.2 (30.6, 45.8)
Any condom distribution at site	28.7 (19.2, 38.3)	63.9 (56.2, 71.5)
Any HIV poster or leaflets distribution	34.4 (24.4, 44.4)	54.7 (46.7, 62.6)
Any health worker outreach	21.9 (13.1, 30.8)	51.7 (43.8, 59.6)
Accept future onsite HIV prevention activities	60.1 (50.1, 70.2)	79.9 (73.0, 86.7)

^a^Weighted proportions

^b^By report of venue informant; all sites were named as places where people go to meet sexual partners by initial community informants

^c^Could not be computed as weighted proportion

### Characteristics of Female Workers at Service and Social Entertainment Venues

A total of 480 women at 14 entertainment venues (12 % of 115 entertainment venues) and 183 women working at 28 service venues (14 % of 200 service venues) were interviewed and tested for syphilis (Table 2). Although the mean age of female workers at both types of venues was similar (24.5 vs. 26.9), entertainment venues employed more women aged 15–19 (30.5 vs. 9.8 %). Workers at service venues were less likely to have finished high school, less likely to live in urban Liuzhou, and more likely to have ever been married. Mean monthly income was nearly identical among entertainment and service venue workers. Service venue workers were more likely to be paid directly by patrons. Most women at both types of venue reported that unemployment, violence, access to health care, AIDS, alcohol abuse, lack of education, and lack of food were big problems in Liuzhou (data not shown). Although few reported injecting drugs, over half of the women reported that injecting drug use was a big problem in Liuzhou. Workers at service venues were five times more likely than workers at entertainment venues to have a positive rapid syphilis test (13.1 vs. 2.4 %). They were also significantly more likely to report risky sexual behaviors including multiple sexual partnerships in the last year (50.5 vs. 17.3 %) and engaging in sex work (45.1 vs. 13.9 %). Women at service venues were more likely to have met a sexual partner at the venue at which they worked (36.6 vs. 6.0 %). However, workers at service venues were also more likely to carry a condom (24.5 vs. 3.2 %) and to have used a condom at last sex (56.3 vs. 29.0 %).

**Table 2:** Characteristics of women who work at entertainment and service venues

	Entertainment venues % (SD or 95 % CI)	Service venues % (SD or 95 % CI)
Number of venues	14	28
Total sample of female workers	480	183
Sociodemographic characteristics		
Mean age	24.5 (23.1, 25.8)	26.9 (25.7, 28.1)
Age 15–19	30.5 (23.6, 37.4)	9.8 (4.5, 15.0)
20–24	37.6 (30.3, 44.8)	33.4 (24.7, 42.0)
25–29	13.8 (8.7, 18.8)	28.5 (20.3, 36.7)
30–34	3.9 (0.9, 6.9)	17.1 (10.0, 24.2)
35–39	4.8 (1.3, 8.2)	5.5 (1.3, 9.6)
40+	9.4 (4.7, 14.2)	5.9 (1.7, 10.0)
Resides in urban Liuzhou	86.9 (83.0, 90.8)	42.7 (35.6, 49.7)
Resides in rural county	13.1 (9.2, 17.0)	57.3 (50.3, 64.4)
Living in Liuzhou < 1 year	38.9 (31.5, 46.3)	46.7 (37.5, 55.8)
Educational attainment < High school	63.2 (56.0, 70.3)	84.1 (77.0, 91.2)
Never married	70.4 (63.4, 77.5)	53.6 (44.4, 62.8)
Has ever been homeless	6.1 (2.5, 9.6)	7.2 (2.4, 12.1)
Has ever been arrested	8.2 (4.8, 11.5)	13.6 (7.5, 19.8)
Employment at venue		
Mean Income in past month all sources (RMB)	1466 (1286, 1645)	1446 (1289, 1604)
Paid by site	86.7 (83.5, 89.9)	59.2 (50.3, 68.0)
Paid by mamie	6.0 (3.7, 8.3)	16.7 (10.0, 23.5)
Paid by patrons	15.7 (12.7, 18.7)	40.0 (31.2, 48.8)
Mean number of days worked in past week	5.9 (5.8, 6.1)	5.9 (5.6, 6.2)
Purchased condom at work in last 4 weeks	11.8 (7.4, 16.3)	23.9 (16.6, 31.1)
Received free condom at work in last 4 weeks	12.6 (8.5, 16.6)	43.0 (34.0, 52.0)
Respondent currently carrying condom (and seen)	3.2 (1.0, 5.3)	24.5 (16.9, 32.0)
Sex work and sexual behavior		
Has ever received gifts or money for sex	13.9 (9.6, 18.3)	45.1 (35.9, 54.3)
Has exchanged sex for money in past 4 weeks	4.9 (2.7, 7.2)	37.2 (28.5, 45.8)
Reported having sex with someone met at venue	6.0 (3.2, 8.7)	36.6 (27.9, 45.3)
Reported 2 or more sexual partners past 12 months	17.3 (12.6, 22.1)	50.5 (41.4, 59.7)
Mean number of sex partners in past 7 days	0.8 (0.6, 0.9)	2.4 (1.8, 3.1)
Syphilis test results and other health risks		
Positive rapid syphilis test	2.4 (0.6, 4.2)	13.1 (7.2, 19.0)
Consumes alcohol at least once per week	22.3 (17.3, 27.3)	14.8 (8.6, 21.0)
Tested for HIV in last 12 months & received results	11.0 (7.1, 14.9)	34.3 (25.7, 43.0)
Condom use at last sex with boyfriend (of those with boyfriend)	33.4 (24.9, 41.9)	54.7 (43.5, 66.0)
Condom use at last sex	29.0 (22.2, 35.8)	56.3 (47.0, 65.6)

Overall, female workers who reported sex work in the past four weeks were more likely to have a positive rapid test for syphilis than women who did not (23.0 vs. 3.0 %); however, 6.9 % of women working at service venues who did not report sex work had a positive rapid test.

### Association Between Working at a Service Venue and Syphilis Infection

We assessed evidence of an association between working at a service venue and having a positive syphilis test (see Fig. 1, Table 3). The crude model estimated that women working at a service venue were more likely to have a positive syphilis test (prevalence ratio = 5.4; 95 % CI 1.4–20.6; prevalence difference = 10.7, 95 % CI 2.1–19.3). After controlling for rural residence, age, and education, women at service venues remained twice as likely to have a positive syphilis test as their peers working in entertainment venues. One explanation for the higher prevalence of syphilis among women at service venues is that they were more likely to have two or more sexual partnerships in the last 12 months, even after controlling for age, education and rural residence (prevalence ratio = 2.6, 95 % CI 1.3–5.2; prevalence difference = 30.3, 95 % CI 10.6–49.0), and women with two or more sexual partnerships in the past 12 months were 5 times more likely to have a positive syphilis test (prevalence ratio: 4.7, 95 % CI 1.6–14.2).

**Table 3:** Outcome of modeling the association between venue type and a positive syphilis test

Models	Prevalence ratio	95 % CI	Prevalence difference	95 % CI
Outcome: Syphilis prevalence ratio/difference of a positive syphilis test at service venues compared with entertainment venues				
Crude	5.4	1.4, 20.6	10.7	2.1, 19.3
Adjusted for urban–rural location	2.2	0.6, 8.2	5.4	0.0–11.6
Adjusted for age, education, and rural	2.3	0.6, 9.2	–^a^	
Outcome: Multiple sexual partnerships prevalence ratio/difference of having 2 or more sexual partnerships in the past year at service venues compared with entertainment venues				
Crude	2.9	1.5, 5.8	33.2	14.0, 52.4
Adjusted for rural location	2.5	1.2, 5.0	27.6	7.9, 47.3
Adjusted for age, education, rural location	2.6	1.3, 5.2	30.3	10.6, 49.9
Outcome: Syphilis prevalence ratio/difference of a positive syphilis test among workers with 2 or more sexual partners in the past year compared to those with fewer than 2 partners				
Crude	6.9	2.1, 23.0	13.8	3.9, 23.7
Adjusted for rural	4.0	1.4, 11.4	3.6	0.0, 7.6
Adjusted for age, education, rural location	4.7	1.6, 14.2	3.7	0.0, 7.7

^a^Model did not converge

## Discussion

We found that service and entertainment venues differed in size, onsite activities, and the male-to-female worker ratio, and that women working at service venues were more likely to have evidence of a current or previous syphilis infection than women working at entertainment venues. Exploration of the association between working at a service venue and syphilis infection showed that women who worked at service venues were more likely to have multiple sexual partnerships, which in turn was strongly associated with having a positive rapid test for syphilis.

This is the first study in a Chinese city to compare the prevalence of a positive rapid syphilis test among women working at entertainment and service venues, regardless of sex worker status. Strengths of this study include the completeness of the sampling frame from which the venues were selected, which encompassed all social and entertainment venues in Liuzhou identified as places where people meet new sexual partners; recruitment of all female workers at selected venues regardless of sex worker status; and the use of verbal informed consent that allowed women to participate without providing their name or other identifying information. Studies that require women to acknowledge sex work as a condition of eligibility, or that collect identifying information, may miss a substantial proportion of women who have greater numbers of new sexual partners but who fear exposure of their behaviors.

Other studies using the PLACE method have found high rates of new sexual partnerships among people at venues identified as places where people meet new sexual partners [24, 25], but this is the first study to assess the difference in syphilis among female venue workers by type of venue. This study contributes to the growing literature in China comparing HIV/STI risk behaviors among service and entertainment venues [3, 15–19, 22, 26, 27]. Surveillance in Guangxi and Yunnan Provinces, respectively, found higher rates of syphilis, HIV, HSV-2, and chlamydia among female sex workers at lower-end venues such as beauty salons compared to those working in higher-end venues such as karaoke bars or nightclubs [3, 16]. Our findings also reinforce results of a 2006 household survey in Liuzhou [13] and of the study of venue patrons reported in this issue [28] indicating that sexual partnerships are often initiated at public venues.

There are several important limitations to the validity and generalizability of our findings. First, the cross-sectional design precludes evidence of a temporal relationship between working at a particular type of venue, having a high rate of new sexual partnerships, and acquiring a sexually transmitted infection. Second, venues were classified as “entertainment” or “service” based on the specific type of venue (e.g., bar, restaurant, or massage parlor) reported by the study staff who visited the venue and conducted interviews there. It is possible that some venues may have been inaccurately categorized, or the venue type may not have been readily identifiable. We included hair salons, massage parlors, and saunas as service venues; others have included these as entertainment venues [14]. Another study found more similarities than we did among massage parlors and nightclubs [29].

Third, feedback from interviewers suggests that women may have underreported sex work at both entertainment and service venues. We expected underreporting of sex work, and suggest that all workers should be provided with health services whether or not they self-report sex work.

In spite of its limitations, the study has important public health implications. Given the high prevalence of infection among workers and the low level of HIV and STI prevention activities at venues found in this study, an extension or scale-up of outreach services at entertainment and service venues is warranted. Venue-based approaches to STI prevention are not new. In China, an intervention consisting of outreach visits to sex workers in entertainment venues in five provinces increased condom use and decreased the prevalence of gonorrhea and chlamydia [30]. Other studies in China have shown that lack of a supportive work environment is associated with increased sexual risk among sex workers [29, 31] and that venue-level support for prevention programs is necessary [14, 32, 33].

Even if it is not feasible to determine which female workers are most at risk at service and entertainment venues, providing interventions to all workers may be an effective strategy for limiting transmission of syphilis and other STIs in China. In our sample, 60 % of the infections among entertainment workers and one-third of the infections among service venue workers occurred among those who did not report sex work. Given the different types of social and sexual risk behaviors found at venues, and the different levels of support for HIV prevention activities, strategies will need to be tailored to the type of venue and the characteristics of the workers.

Although the percentage of women with a positive syphilis test was higher at service venues, prevention programs should not exclude women at entertainment venues, which are larger and employ more women, and where condom use and availability were found to be dramatically lower. Based on the number of venues identified in Liuzhou where people meet new sexual partners and the average number of workers at sampled venues, we estimate that approximately 4,274 women work in entertainment venues and 2,794 work in service venues in Liuzhou.

The study suggests that additional research into the factors associated with recruitment, retention, and departure of female workers into service and entertainment venues might identify additional strategies for understanding sexual risk dynamics at these venues and reducing the risk of women during their employment.

## Conclusions

Although this study misses some important groups of women at risk of acquiring syphilis who do not work at entertainment or service venues, including sex workers who solicit from the streets or through the internet, this is the first study in a Chinese city to compare the prevalence of a positive rapid syphilis test among women working at entertainment and service venues, regardless of sex worker status. These findings reinforce prior evidence that sexual partnerships are often initiated at public venues and that public health outreach to venues where people meet new sexual partners is warranted.
